# Mechanisms of *Borrelia burgdorferi* internalization and intracellular innate immune signaling

**DOI:** 10.3389/fcimb.2014.00175

**Published:** 2014-12-15

**Authors:** Tanja Petnicki-Ocwieja, Aurelie Kern

**Affiliations:** Division of Geographic Medicine and Infectious Diseases, Tufts Medical CenterBoston, MA, USA

**Keywords:** *Borrelia burgdorferi*, Lyme disease, Toll-like receptor signaling, Nod-like receptor signaling, phagocytosis, endosomal signaling

## Abstract

Lyme disease is a long-term infection whose most severe pathology is characterized by inflammatory arthritis of the lower bearing joints, carditis, and neuropathy. The inflammatory cascades are initiated through the early recognition of invading *Borrelia burgdorferi* spirochetes by cells of the innate immune response, such as neutrophils and macrophage. *B. burgdorferi* does not have an intracellular niche and thus much research has focused on immune pathways activated by pathogen recognition molecules at the cell surface, such as the Toll-like receptors (TLRs). However, in recent years, studies have shown that internalization of the bacterium by host cells is an important component of the defense machinery in response to *B. burgdorferi*. Upon internalization, *B. burgdorferi* is trafficked through an endo/lysosomal pathway resulting in the activation of a number of intracellular pathogen recognition receptors including TLRs and Nod-like receptors (NLRs). Here we will review the innate immune molecules that participate in both cell surface and intracellular immune activation by *B. burgdorferi*.

## Introduction

Lyme disease is caused by the spirochete *Borrelia burgdorferi* transmitted through a tick bite. The disease manifests as early localized skin inflammation (erythema migrans) occurring at the site of the tick bite. Late stage disease is characterized by inflammation of the heart, the joints, the nervous system or the skin. The incidence of human infections has risen steadily over the last 15 years and Lyme disease is the most common tick-borne disease in the United States and Europe. More than 30,000 cases are reported annually in the US and the number of cases is estimated around 85,000 yearly in Europe (Lindgren et al., 2006; Centers for Disease Control and Prevention, 2013).

Innate immune responses are the first responders to infection and the catalyst of inflammation causing much of Lyme disease pathology. Recently, studies have shown that phagocytosis plays a role in initiating inflammatory responses (Moore et al., 2007; Shin et al., 2008; Salazar et al., 2009). Because of the requirement for pathogen internalization, much interest has been generated in studying intracellular signaling pathways. As a result, in addition to pathogen recognition by Toll-like receptors (TLRs), intracellular receptors such as the NOD-like receptors (NLRs) have been shown to participate in *B. burgdorferi* signaling. These families of receptors do not act in isolation and there is considerable cross talk among the innate immune signaling pathways activated (Takeuchi and Akira, 2010). TLR2 has been shown to initiate a significant portion of the inflammatory output in response to *B. burgdorferi*. The TLR2 ligands are *Borrelia* cell surface lipoproteins, the best-characterized being Outer Surface Protein A (OspA) (Hirschfeld et al., 1999; Lien, 1999; Takeuchi and Akira, 2010).

Cellular compartmentalization is increasingly recognized as having a significant role in the regulation of innate immune signaling. Although this concept has been broadly understood as distinguishing between cell surface receptors, such as TLRs 2, 4, and 5 and intracellular sensors, such as TLRs 3, 7, and 9, as well as NLRs, it has recently become apparent that intracellular trafficking to different sub-cellular compartments and organelles, such as mitochondria and peroxisomes, plays a more intricate role in innate immune regulation than previously thought (Eisenbarth and Flavell, 2009; Blasius and Beutler, 2010; Dixit et al., 2010; Kagan, 2012). Here we will review various intracellular innate immune pathways activated by *B. burgdorferi* and how they may collectively contribute to inflammatory signaling.

## Toll-like receptors

### Cell surface signaling

#### Recognition of B. burgdorferi at the cell surface

Due to the importance of bacterial phagocytosis into host cells, significant research in recent years has been devoted to understand the involvement of cell surface molecules in the internalization of *B. burgdorferi*. Spirochete internalization involves attachment or tethering of the bacteria to the host cell followed by engulfment into the host cell. These two processes, although linked, often involve different sets of cells surface molecules. *B. burgdorferi* contains a number of molecules known to function as adhesins and participate in the attachment of the bacterium to the host cell. One group of receptors shown to participate in tethering of *B. burgdorferi* to the cell surface is the integrin family, specifically integrins α_v_β_3_, α_5_β_1_,and α_M_β_2_(CD18/CD11b, Mac-1, CR3) (Cinco et al., 1997; Coburn et al., 1998). Integrin α_v_β_3_has been shown to bind to the p66 protein of *B. burgdorferi*, but has not been shown to play a direct role in the initiation of immune responses (Coburn et al., 1998; Coburn and Cugini, 2003). It does, however, participate in adherence and potentially internalization of the bacterium into the host cell. Interestingly, integrin α_v_β_3_ has been suggested to tether TLR2 ligands to the host cell via interaction with the serum protein vitronectin (Gerold et al., 2008). This has not been explored in the context of *B. burgdorferi* adhesion. Integrin α_M_β_2_ has been shown to participate in the attachment of *B. burgdorferi* to the cell surface (Cinco et al., 1997) and together with the TLR2 associated GPI anchored receptor, CD14, α_M_β_2_ mediates the internalization of the bacterium into the host cell (Hawley et al., 2012).

#### Signal transduction from the cell surface

Signaling from the plasma membrane is a necessary component of the *B. burgdorferi* response, the mechanisms of which remain to be completely described. Understanding how cell surface accessory molecules contribute to the recognition of the ligand by the TLR is an active area of research. In the model of TLR4 signaling, integrin α_M_β_2_ participates in the recruitment of the Toll-interleukin 1 receptor (TIR) domain-containing adapter protein (TIRAP) to Phosphatidylinositol 4,5-bisphosphate (PIP2) rich membranes, where TIRAP interacts with PIP2 and initiates cells surface signaling by recruiting the MyD88 signaling adaptor (Kagan and Medzhitov, 2006). TLR2 is the only other TLR that has been shown to utilize TIRAP for signaling and thus is likely that interaction with PIP2 is also important for TLR2 signaling (Yamamoto et al., 2002). However, there is significant interest in uncovering the signaling mechanism behind the ability of α_M_β_2_ to traffic ligands and the TLR receptor to PIP2 rich locations and recruit signaling molecules.

*B. burgdorferi* does not contain lipopolysaccharide (LPS) and does not activate TLR4 (Takayama et al., 1987; Berende et al., 2010). However, it contains other ligands that activate different TLRs. Specifically, *B. burgdorferi* activates TLR2/1 heterodimers through recognition of the triacylated lipid moiety on its cell surface localized lipopeptides (Hirschfeld et al., 1999; Alexopoulou et al., 2002). *B. burgdorferi* has been shown to activate TLR5, potentially through the spirochetal flagellin (Shin et al., 2008). Recruitment of MyD88 to the plasma membrane by TIRAP for TLR2 signaling or directly to TLR5 results in a signaling cascade which proceeds through the recruitment of the IRAK kinases, the E3 ubiquitin ligase, TRAF6, and TAK1 leading to the activation of MAP kinases and of NF-kB through the IKK complex (Takeda and Akira, 2004). This signaling pathway leads to the activation of pro-inflammatory cytokines such as IL-6, IL-12, TNF-α, and pro-IL1β. Type I IFNs have not been shown to be activated from this signaling cascade initiated at the plasma membrane.

### Intracellular signaling

#### Internalization of B. burgdorferi into host cells

Although there are a number of cell surface molecules that mediate phagocytosis of *B. burgdorferi*, the signaling events during the process of internalization are under investigation. Early studies into the mechanisms behind *B. burgdorferi* internalization indicate that it occurs through coiling, rather than conventional, phagocytosis, in which the bacteria attach to the host cell surface and are rolled into a single fold of the plasma membrane (Rittig et al., 1992). In studies with primary human macrophages it was shown that interaction with the bacteria leads to the formation of f-actin rich structures. The actin polymerization is mediated by the Wiskott-Aldrich syndrome family protein (WASP) and Arp2/3 complex, particularly during integrin α_M_β_2_and Fcγ receptor mediated phagocytosis (Linder et al., 2001; Shin et al., 2009). The regulatory pathways influencing actin polymerization and internalization of *B. burgdorferi* are mediated by the small GTPases Cdc42 and Rac1 (Linder et al., 2001). In addition, PI3K signaling was shown to be required for phagocytosis of *B. burgdorferi* by murine macrophages (Shin et al., 2009).

Although some cell surface molecules may serve as tethers, other molecules seem to have a more direct role in internalization. As described, CD14 is a molecule that mediates endocytosis of *B. burgdorferi* (Hawley et al., 2012). However, this is in itself a confusing finding in that CD14 is not known to have any cytoplasmic signaling domains that could initiate a signaling cascade leading to phagocytosis. CD14 has been shown to bind integrin α_M_β_2_ and localize it to lipid rafts and it is possible that it then interacts with other molecules that can mediate endocytosis (Hawley et al., 2013). The primary role of this integrin seems to be in the attachment of the *B. burgdorferi*, rather than its internalization, and the signaling pathways activated by the integrin to mediate phagocytosis have not been described in the context of *B. burgdorferi*. Interestingly, integrin α_3_β_1_ has been shown to mediate *B. burgdorferi* and TLR2/1 ligand signaling (Marre et al., 2010). However, unlike α_M_β_2_, α_3_β_1_ was not shown to mediate the attachment of *B. burgdorferi* or other TLR2 ligands to the cell surface but rather to participate in internalization (Behera et al., 2006; Marre et al., 2010). Overall, the β_1_integrin is required for internalization of *B. burgdorferi* into murine fibroblasts and to utilize the Src kinase signaling pathway for internalization (Wu et al., 2011). It is unclear if α_3_β_1_directly activates the Src signaling cascade, nor if CD14 and integrin α_3_β_1_ cooperate to mediate phagocytosis of *B. burgdorferi*.

The complexity of molecule involvement in the phagocytic process is increased by the added role of scavenger receptors, which have also been shown to participate in TLR2 and *B. burgdorferi* signaling. CD36, a type B scavenger receptor, has been shown to be important for the internalization of TLR2 ligands and numerous studies have been devoted to understanding the mechanisms behind CD36 cooperation with TLR2. However, its role in *B. burgdorferi* internalization has not been explored (Shamsul et al., 2010). The scavenger receptor Macrophage Receptor with Collagenous Structure (MARCO), which plays a role in the internalization of a variety of microbial ligands, does mediate *B. burgdorferi* phagocytosis. In our studies we showed that MARCO was significantly up-regulated upon *B. burgdorferi* stimulation. The up-regulation of MARCO was dependent on MyD88 and MARCO deficient macrophage showed a decrease in the phagocytosis of *B. burgdorferi* (Petnicki-Ocwieja et al., 2013). These findings offered one possible explanation for the partial phagocytic defect previously observed in MyD88 deficient macrophage (Shin et al., 2008; Petnicki-Ocwieja et al., 2013). It is yet unclear if MARCO participates in *B. burgdorferi* attachment to the cell surface. Significant work still needs to be done to determine how these cell surface accessory molecules: integrins, scavenger receptors and the GPI anchored receptor, CD14, cooperate to mediate signaling and internalization of *B. burgdorferi* into the host cell.

#### Signal transduction from the endosomal compartment

TLR signaling can be initiated from both the plasma membrane and intracellular compartments. TLR5 has not been shown to signal from intracellular compartments and thus will not be discussed further in this context. Interestingly, we and others have recently shown that in addition to being localized at the plasma membrane, TLR2 mediates signaling from endosomal vesicles in response to *B. burgdorferi* and TLR2 synthetic ligands. Inhibition of endosomal acidification upon *B. burgdorferi* stimulation results in a decrease in type I IFN and pro-inflammatory cytokine activation, such as IL-6 (Marre et al., 2010; Cervantes et al., 2011). TLR2 cooperates with other endosomal TLRs to generate a *B. burgdorferi* specific inflammatory response. *B. burgdorferi* activates TLR7/8 and TLR9, which are endosomally localized TLR receptors (Shin et al., 2008; Petzke et al., 2009; Cervantes et al., 2011, 2013). Adaptor molecules, such as MyD88, are recruited to the endosomal compartment to transduce signals for the activation of inflammatory cytokines and type I IFN from these endosomal TLRs (for a detailed review on TLR 7/8 and 9 in *B. burgdorferi* signaling, please see Cervantes, Hawley and Salazar in this issue).

The localization of TLR2 at two different cellular locations requires that signaling molecules are able to distinguish the cellular localization of TLRs and assemble cell location specific signaling complexes. The only other TLR that has been shown to signal from two different cellular locations is TLR4. In the model of TLR4 signaling, signaling pathways from the cell surface vs. the endosome are clearly distinguished. TLR4 mediates signaling from the plasma membrane via TIRAP/MyD88 for MAP kinase and NF-kB activation resulting in pro-inflammatory cytokine activation. From the endosome TLR4 utilizes an entirely different set of adaptors, TRAM and TRIF, which are the signaling platform used to signal for type I IFNs, although TRAM/TRIF also mediate a delayed wave of NF-kB and pro-inflammatory cytokine activation (Kawai and Akira, 2011).

Investigating the TLR2 signaling complex at the plasma membrane in comparison to the endosome, we found that TLR2 was also able to utilize the adaptor TRIF. TRIF deficient macrophage showed a reduction in type I IFN activation and secretion of IL-6 (Petnicki-Ocwieja et al., 2013). This was an unexpected result as TRIF was previously thought not to participate in TLR2 signaling. Interestingly, as opposed to the clear separation of signaling pathways in the TLR4 model, TLR2/TRIF signaling was dependent on MyD88, suggesting that the MyD88 and TRIF signaling pathways were interconnected. In addition to participating in TLR7/8 and 9 signaling, MyD88 may also participate in TLR2 signaling at the endosome. *In vivo*, TRIF deficient mice did not show any deficiencies in the ability to control bacterial loads in the joints of *B. burgdorferi* infected mice in comparison to wild type mice (Petnicki-Ocwieja et al., 2013). However, TRIF deficient mice did have increased levels of inflammatory cytokines in the joints, suggesting that TRIF has an important role in controlling immune responses leading to inflammation but not responses leading to control of pathogen burden. This might in part be due to the fact that, unlike MyD88 deficient cells, TRIF deficient cells do not have any observable phagocytic defects.

Intracellular activation of immune pathways has been extensively studied in the case of viral infections. The intracellular activation of type I interferons (IFNs) was for a long time considered to be strictly a viral response. Recently, type I IFN activation has been shown to play an important role in a large number of bacterial infections (Katze et al., 2002; Perry et al., 2005). In *B. burgdorferi* infection, the type I IFN response has also been shown to be important for the development of murine Lyme arthritis (Miller et al., 2008; Petzke et al., 2009; Salazar et al., 2009; Cervantes et al., 2011). Type I IFN activation initiated by TLRs is mediated by interferon regulatory factors (IRFs). In studies with TLR2 signaling, IRF1, and IRF7, both of which have been shown to bind MyD88, participate in TLR2 signaling (Dietrich et al., 2010). Downstream of the adaptor TRIF, TRAF3 is responsible for localizing an IRF3 signaling complex to the endosome leading to type I IFN activation. *B. burgdorferi* stimulation has also been shown to proceed through IRF7 via TLR2 and TLR7 and 9 (Petzke et al., 2009; Petnicki-Ocwieja et al., 2013). Interestingly, studies have also shown that IRF3 is required for the type I IFN response to *B. burgdorferi* (Miller et al., 2010).

## Nucleotide binding oligomerization domain receptors (NOD-like receptors)

TLRs sense the extracellular and the endosomal compartments whereas RIG-like receptors (RLRs) and Nod-like receptors (NLRs) are intracellular sensors. In addition to TLRs, NLRs also participate in *B. burgdorferi* mediated intracellular signaling. From the receptors in the NLR family, Nod1, and 2 and the inflammasome complex are the best studied.

### Nod1 and Nod2

Nod1 and Nod2 multi-domain proteins in the NLR family and are involved in the recognition of intracellular pathogens. NLRs contain an N-terminal effector domain which is thought to participate in protein-protein interactions with downstream signaling molecules, a central nucleotide-binding oligomerization domain (NBD or NACHT), and a C-terminal leucine-rich repeat domain for ligand recognition (LRR). Members of the NLR family can be sub-divided based on their N-terminal domain, which can be a Caspase Recruitment Domain (CARD), a pyrin domain (PYR), or a baculovirus IAP repeat domain (BIR) (Kanneganti et al., 2007) (Wilmanski et al., 2007). Many NLRs are complexed into the inflammasome, a signaling platform which activates caspase-1. Others, such as Nod1 and Nod2 are not considered to be inflammasome components and instead recruit the RIP2 kinase (or RICK) through a CARD-CARD interaction leading to the expression of pro-inflammatory cytokines (Moreira and Zamboni, 2012). Nod1 and Nod2 cytoplasmic receptors sense intracellular bacteria through muropeptides derived from bacterial peptidoglycans although some data suggest they are also able to sense viruses and protozoan parasites (Strober et al., 2005). *In vitro* stimulation of Nod2 deficient macrophage with *B. burgdorferi* results in a decrease in pro-inflammatory and type I IFN induction, suggesting Nod2 plays a role in the activation of inflammatory signals (Sterka and Marriott, 2006; Chauhan et al., 2009; Oosting et al., 2010a; Petnicki-Ocwieja et al., 2011). Nod1 does not seem to play a significant role in *B. burgdorferi* signaling.

In contrast to the pro-inflammatory effect of Nod2 *in vitro*, the opposite pattern is observed *in vivo*. Nod2 deficiency results in increased inflammation in the heart and joints after *B. burgdorferi* infection *in vivo.* This *in vivo* finding suggests that Nod2 is also involved in the suppression of the inflammatory signal during *B. burgdorferi* infection (Petnicki-Ocwieja et al., 2011). Nod2 has been extensively studied in other *in vivo* inflammation models and in some systems, Nod2 deficiency results in increased rather than decreased pathology, as we have observed for *B. burgdorferi*. Such cases include chronic *M. tuberculosis* infection (Gandotra et al., 2007) and the Inflammatory Bowel Disorder–Crohn's disease, to which Nod2 mutations have been genetically linked (Hugot et al., 2001; Netea et al., 2004). A possible explanation for our *in vivo* observations may lie in the long-term nature of the infection. Prolonged stimulation of Nod2, similar to endotoxin/LPS mediated tolerance, results in the downregulation of pro-inflammatory cytokines in the intestinal environment, thus maintaining homeostasis (Hedl et al., 2007). In the context of *B. burgdorferi* infection, we hypothesized that Nod2 might play a role in controlling over exuberant inflammation *in vivo*, possibly via a mechanism of tolerance through stimulation of Nod2 or other PRRs (Petnicki-Ocwieja et al., 2013). Thus, Nod2 may have roles in both resistance to microorganisms and also in tolerance to microbial stimulation. The signals, which lead Nod2 to switch from its inflammatory role to its tolerance function, remain unknown.

### Inflammasome

Inflammasomes, described in 2002 (Martinon et al., 2002), are multi-protein complexes that recognize diverse stimuli such as pathogen-associated molecular patterns (PAMPs) and damage-associated molecular patterns (DAMPs). These complexes are formed in response to inflammation and control the production of pro-inflammatory cytokines especially interleukin-1β (IL1β) and interleukin-18 (IL18). Their formation is initiated in the cell cytosol and comprises a NLR receptor, an adaptor protein ASC (Apoptosis-associated Speck-like protein containing a C-terminal CARD) and the cysteine protease caspase-1. The recruitment of caspase-1 to the inflammasome leads to its proteolytic activation. The activated caspase cleaves pro-IL1β and pro-IL18 into their active secreted form (Couillin et al., 2011; Strowig et al., 2012). Caspase-1 is also known to be involved in the activation of the NF-kB pathway resulting in expression of other pro-inflammatory cytokines and in cell death. Among the inflammasomes, the NLRP3 complex is the best characterized and has been looked at in the context of *B. burgdorferi*, although no significant inflammatory phenotype *in vivo* was observed (Eisenbarth and Flavell, 2009; Oosting et al., 2012). NLRP3 has been shown to recognize a large number of microbial and non-microbial stimuli (MDP, nucleic acids, toxins, silica, urate crystals etc.) but other proteins of the NLR family that recognize flagellin (NLRC4/IPAF) might also play a role (Franchi et al., 2010).

Clinical manifestations of Lyme disease can involve inflammation of the skin, joints and heart (Steere, 2001). Cytokine levels measured in erythema migrans lesions from Lyme patients have revealed the presence of IFNγ but also IL1β and IL6 (Müllegger et al., 2000). In addition, IL1β and IL18 levels are elevated in serum of patients presenting Lyme arthritis (Pietruczuk et al., 2006). *In vitro* it has been shown that IL1β is secreted by human PBMC after phagocytosis of live *B. burgdorferi* (Cruz et al., 2008). Despite the presence of cytokines that are the result of inflammasome activation, the role of the inflammasome in the host immune response to *B. burgdorferi* is not clear.

Mice deficient in caspase-1 and ASC, had no effect on host defense against *B. burgdorferi in vivo* (Liu et al., 2009). However, Oosting et al. demonstrated that *B. burgdorferi* activates the inflammasome and the production of IL-17 through a caspase-1-dependent mechanism in an *in vivo* model of Lyme arthritis (Oosting et al., 2010b). Both studies were done in knockout mice that were later described as double knockout for caspase-1 and caspase-11, suggesting that some of the phenotypes observed may be attributed to the deficiency in caspase-11 (Kayagaki et al., 2012). Recently a paper using mice lacking only caspase-1 showed that acute Lyme arthritis seems to be dependent on the adaptor ASC and caspase-1 (Oosting et al., 2012). These different results regarding the role of caspase-1 and ASC may be due to the fact that the infection models used in the studies differed in the route of inoculation and the time points examined. As a result, the role of the inflammasome in *B. burgdorferi* infection remains unclear. One hypothesis that may resolve some of the discrepancies is that the inflammasome may have a different role during acute and chronic phases of infection.

## Other intracellular pathways

While studies have found that the activation of type I IFNs is driven by endosomal TLRs and Nod2, other studies have found that type I IFN signaling is TLR and Nod2 independent (Miller et al., 2008). Although differences in the types of knockout cells or activation state of the macrophage are possible explanations for such discrepancies, it is likely that at least part of the type I IFN response generated occurs via other intracellular sensors. A recent study suggests that TLR independent type I IFN signals may be responsible for naïve B cell accumulation in Lyme disease (Hastey et al., 2014). The involvement of members of the RLR family, such as the dsRNA receptors RIG-1 or MDA5, or the DNA-sensing AIM2 inflammasome complex, remains to be explored in the context of *B. burgdorferi* infection. Alternatively, it is possible none of the above mentioned sensors may be responsible for a type I IFN response. Miller et al. showed in their studies that supernatants from log phase cultures of *B. burgdorferi* were still able to induce IFN gene-related transcripts even when the supernatants were filtered and treated with DNase and RNase, excluding recognition by RLRs or AIM2 (Miller et al., 2010). Thus, it is possible that there is a soluble and possibly secreted *B. burgdorferi* non-nucleic acid ligand that activates type I IFNs via IRF3. In this case, both the sensor and the ligand remain to be identified.

## Conclusions

**Figure 1:**
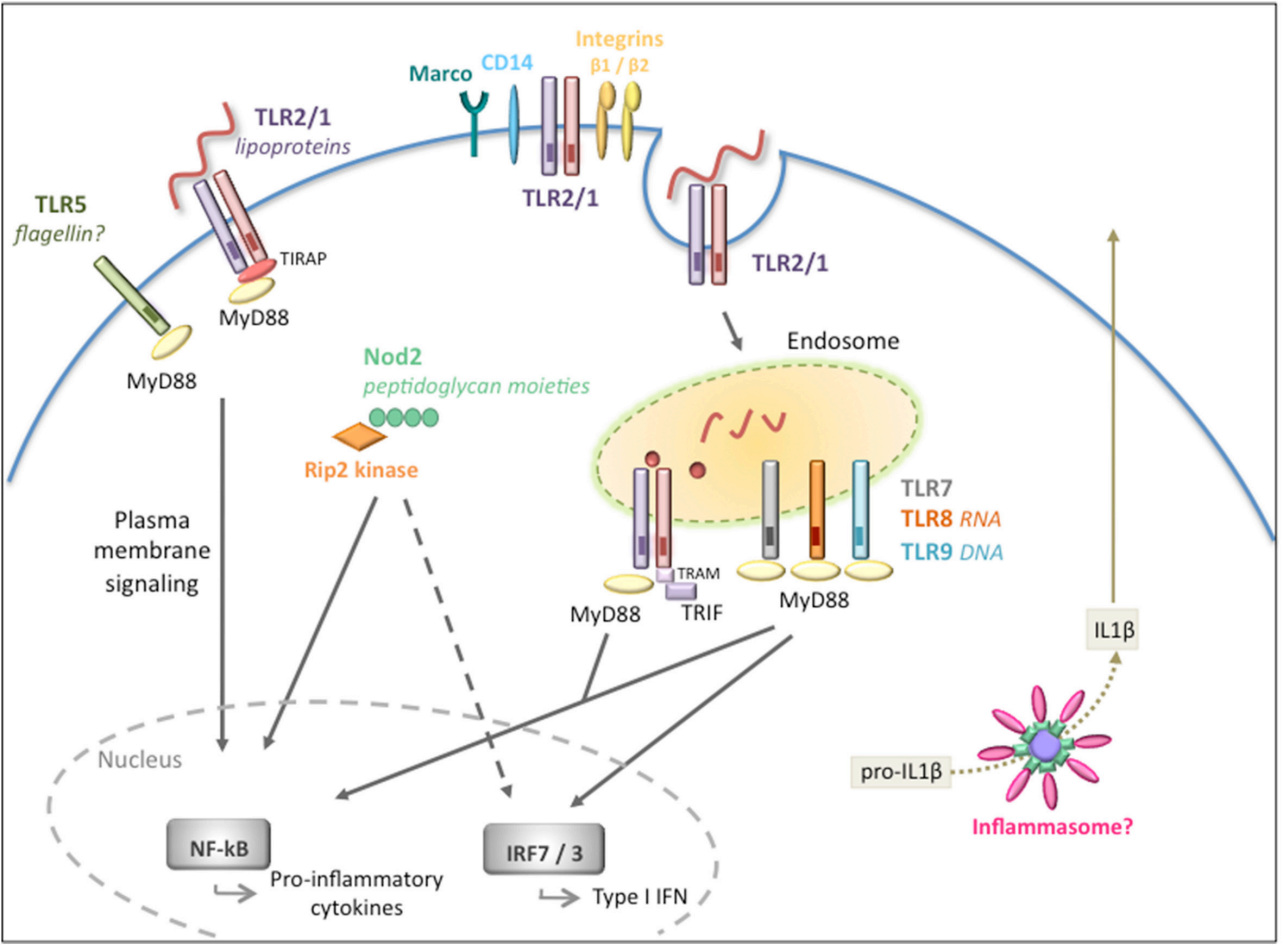
**Extracellular and intracellular signaling pathways mediated by *B. burgdorferi***. Overview of the pathogen recognition receptors (PRRs) involved in the recognition and signaling in response to *B. burgdorferi*. Cell surface signaling is primarily mediated by TLR2/1 leading to pro-inflammatory cytokine production. Integrins and CD14 are known to recognize and internalize the spirochete but their role in intracellular signaling is not fully understood. Intracellular receptors located at the endosome, in particular TLR2/1, TLR7/8, and TLR9, are activated by different *B. burgdorferi* ligands and recruit adaptors such as MyD88 and/or TRIF to transduce signals for the activation of inflammatory cytokines and type I IFNs. The Nod2 receptor also plays a role in recognition of *B. burgdorferi* and in the induction of inflammatory responses, but it might have a dual regulatory role depending on the stage of infection. The inflammasome is likely to be involved, however, *in vivo*, it is unclear whether the inflammasome is required for the development of host responses to the pathogen.

The innate immune system rapidly responds to the presence of pathogens. Although there are a number of receptors localized at the cell surface that detect invasive pathogens before they enter the cells, there is also an entire network of “second-wave” intracellular receptors that alert the host to a microbial presence. Thus, immune responses to microbial invasion are comprised of a signaling network involving multiple receptors (Figure 1).

The contribution of a number of these intracellular receptors to pathogen infection may not be in the aspect of bacterial clearance but rather in exacerbating the inflammatory response. We have seen evidence of this in mice deficient in Nod2 and TRIF, which do not affect pathogen burden but do affect severity of inflammation (Petnicki-Ocwieja et al., 2011, 2013). Treatment of Lyme arthritis requires the use of antimicrobials to clear the pathogen (Wormser et al., 2006). However, due to the fact that much of the pathology is a result of excessive inflammation, there could be a clinical application for immunomodulatory drugs. Thus, understanding intracellular signaling pathways engaged by *B. burgdorferi* could lead to the development of anti-inflammatory treatments that, when necessary, could be used in combination therapy for Lyme arthritis.

One receptor we have focused on is TLR2, which has been shown to be responsible for a significant portion of *B. burgdorferi* initiated pro-inflammatory signaling. However, studies have shown that other TLRs, such as TLR7/8 and TLR9 also signal for the activation of inflammatory responses, including type I IFNs (Petzke et al., 2009; Salazar et al., 2009; Cervantes et al., 2011, 2013). Futhermore, TLR2 and TLR8 were shown to cooperate in response to *B. burgdorferi* (Cervantes et al., 2011). In addition, Nod2 has also been shown to play a role in mediating some of the *B. burgdorferi* inflammatory response. Thus, the *B. burgdorferi*-induced inflammatory signature is a result of contribution of multiple receptors.

It is unclear how the cell directs this signaling network. In recent years it has been shown that localization to different cellular compartments plays a significant role in coordinating the activation of innate immune pathways. The broad classification of cell surface being distinct from intracellular receptors is no longer sufficient when describing the complexity of signaling pathways. Although “intracellular” is used to broadly imply endosomal localization it is now known that immune signaling can be orchestrated from intracellular vesicles and organelles (Kagan, 2012). In addition, some immune signaling complexes previously thought to be strictly cytoplasmic may in fact be recruited to specific compartments containing ligands. In support of this hypothesis, recent data has shown that caspase-1 can accumulate at the phagosome, suggesting that molecules of the inflammasome are also recruited to phagosomal compartments. In the *B. burgdorferi* model, we have made strides in understanding the cellular distribution of different receptors at the cell surface as well as inside the host cell. However, the role of vesicular trafficking to different compartments in the *B. burgdorferi* host response and *B. burgdorferi* pathogenesis remains to be explored.

### Conflict of interest statement

The authors declare that the research was conducted in the absence of any commercial or financial relationships that could be construed as a potential conflict of interest.
